# *NOS3* rs3918188C>A is associated with susceptibility to resistant hypertension while *CES1* genetic variation was not associated with resistant hypertension among South Africans

**DOI:** 10.3389/fgene.2025.1608423

**Published:** 2025-06-04

**Authors:** Jonathan N. Katsukunya, Revina Naicker, Nyarai D. Soko, Dirk Blom, Phumla Sinxadi, Emile R. Chimusa, Brian Rayner, Erika Jones, Collet Dandara

**Affiliations:** ^1^ Division of Human Genetics, Department of Pathology and Institute of Infectious Disease and Molecular Medicine (IIDM), Faculty of Health Sciences, University of Cape Town, Cape Town, South Africa; ^2^ SAMRC/UCT Platform for Pharmacogenomics Research and Translation, South African Medical Research Council, Cape Town, South Africa; ^3^ Department of Pharmaceutical Technology, School of Allied Health Sciences, Harare Institute of Technology, Harare, Zimbabwe; ^4^ Division of Lipidology and Cape Heart Institute, Department of Medicine, Groote Schuur Hospital and Faculty of Health Sciences, University of Cape Town, Cape Town, South Africa; ^5^ Division of Clinical Pharmacology, Department of Medicine, Groote Schuur Hospital and Faculty of Health Sciences, University of Cape Town, Cape Town, South Africa; ^6^ Department of Applied Sciences, Faculty of Health and Life Sciences, Northumbria University, Newcastle, United Kingdom; ^7^ Division of Nephrology and Hypertension, Department of Medicine, Groote Schuur Hospital and Faculty of Health Sciences, University of Cape Town, Cape Town, South Africa

**Keywords:** *CES1*, *NOS3*, pharmacogenomics, hypertension, Africans, ACE inhibitors, enalapril

## Abstract

**Introduction:**

Genetic variation in genes coding for enzymes metabolising antihypertensive drugs, may affect the efficacy of angiotensin converting enzyme (ACE) inhibitors such as enalapril, potentially leading to resistant hypertension (RHTN). We set out to evaluate the contribution of genetic variation in *CES1* and *NOS3* genes on susceptibility to RHTN, as well as estimate the frequencies of *CES1* copy number variation (CNV) in African and Mixed Ancestry (MA) populations of South Africa.

**Methods:**

Using a retrospective age, sex and ethnicity matched case-control study design, 379 participants with hypertension belonging to the African and MA ethnic groups were recruited. Cases were participants with RHTN (i.e., blood pressure (BP) ≥140/90 mmHg on ≥3 antihypertensive drugs or BP < 140/90 mmHg on >3 antihypertensive drugs, including a diuretic). Cases were matched to controls with similar characteristics (age (±5 years), sex and ethnicity) in a 1:1 ratio. Controls were participants with hypertension that was under control (BP < 140/90 mmHg on ≤3 antihypertensive drugs). Five polymorphisms in *CES1* and *NOS3* were characterized using polymerase chain reaction-restriction fragment length polymorphism (PCR-RFLP), quantitative PCR and validated using Sanger sequencing. The additive model of inheritance and multivariable logistic regression were used to determine associations between genotypes and RHTN while adjusting for potential confounding variables.

**Results and discussion:**

*NOS3* rs3918188A/A (aOR: 0.13; CI: 0.04–0.41; P = 0.0009) genotype and *NOS3* rs2070744–rs1798883–rs3918188G–T–A haplotype (OR: 0.54; CI: 0.37–0.78; P = 0.001) appeared to confer protection against RHTN among MA participants only. *CES1* rs2244613C>A and *CES1* CNV were not significantly associated with RHTN. However, there appeared to be quantitative differences in *CES1* CNV profiles across ethnic groups. We speculate that *NOS3* rs3918188A allele may affect *NOS3* gene expression, potentially leading to increased amounts of the vasodilator, nitric oxide (NO) and favourable outcomes in individuals taking antihypertensives drugs such as enalapril.

**Conclusion:**

*NOS3* genetic variation seems important in the susceptibility to RHTN among Africans and requires further studies.

## Introduction

Hypertension is a serious public health concern, affecting over 1 billion people globally (WHO, 2021). It is a major contributor to the population attributable risk for stroke, myocardial infarction and heart failure, which are among the leading causes of death worldwide (Olsen et al., 2016; Zhou et al., 2021). Resistant hypertension (RHTN) is defined as uncontrolled blood pressure (BP) (≥140/90 mmHg) in an adherent patient taking three or more antihypertensive drugs including a diuretic, or controlled BP (<140/90 mmHg) in an adherent patient taking four or more antihypertensive drugs (Dudenbostel et al., 2016). RHTN affects up to 21% of hypertensive patients globally (Acelajado et al., 2012; Jones et al., 2017), between 4%–19% of hypertensives in Africa (Nansseu et al., 2016), and nearly 13% of South Africans with hypertension (Moosa et al., 2016).

Angiotensin converting enzyme (ACE) inhibitors are one of the most frequently used class of antihypertensive drugs in patients with hypertension, including those with RHTN (Jones et al., 2020; Unger et al., 2020). Enalapril is the preferred ACE inhibitor in the Essential Drug List (EDL) of South Africa and hence the second most prescribed antihypertensive drug in patients attending a tertiary-level Hypertension Clinic at Groote Schuur Hospital, in South Africa (Soko et al., 2023). Response to enalapril has been reported to vary widely among individuals and its use is associated with several potential adverse reactions, such as angioedema, dry-cough, hypotension and hyperkalaemia (Moholisa et al., 2013; Katsukunya et al., 2023). Several enzymes are involved in the pharmacokinetics and pharmacodynamics of enalapril including carboxylesterase-1 (CES1) and endothelial nitric oxide synthase (NOS3/eNOS) respectively. As such, genetic variation in *CES1* and *NOS3* genes has been reported to influence treatment outcomes in individuals taking enalapril (Oliveira-Paula et al., 2019) and may potentially contribute to susceptibility to RHTN.

*CES1* gene is located on chromosome 16q12.2, has 14 exons and is ∼33 kb long and encodes for the CES1 enzyme, a serine esterase, responsible for the conversion of enalapril to enalaprilat. Enalaprilat is up to 20 times more potent in lowering BP than enalapril, thus, this conversion is essential for enalapril efficacy (Davis et al., 2007). *CES1* is highly polymorphic, has a complicated structure and is subject to segmental duplications resulting in copy number variations (Yoshimura et al., 2008). Polymorphisms such as *CES1* rs2244613C>A and copy number variation (CNV) have been shown to affect the pharmacokinetics of ACE inhibitors, including enalapril. However, studies report inconsistent effects (Ikonnikova et al., 2022a; Wang et al., 2016; Her et al., 2021; Stage et al., 2017a). Recently, *CES1* rs2244613C allele carriers were reported to present with reduced peak and trough concentrations of both enalapril and enalaprilat compared to rs2244613A/A genotype carriers in European patients with hypertension (Ikonnikova et al., 2022b).

The *NOS3* gene, located on chromosome 7q36.1, has 28 exons and encodes for endothelial nitric oxide synthase (eNOS/NOS3), a 1203 amino acid long protein. According to Oliviera-Paula and colleagues (Oliveira-Paula et al., 2017), ACE-inhibition is not the only mechanism of action of ACE-inhibitors and upregulation of NOS3 activity, stimulating the production of increased nitric oxide (NO), a vasodilator (Oliveira-Paula et al., 2016a) also contributes to BP lowering. Several polymorphisms in *NOS3* have been reported in connection with hypertension and antihypertensive drug response. For example, *NOS3* rs2070744C>T and rs1799983T>G have been shown to affect susceptibility to essential hypertension (Gamil et al., 2017; Jemaa et al., 2007; Amrani-Midoun et al., 2019; Nassereddine et al., 2018) and response to enalapril (Masilela et al., 2021) whereas *NOS3* rs3918188C>A has been reported to be associated with enalapril response among Brazilians, with *NOS3* rs3918188A allele carriers showing lower decreases in BP (Oliveira-Paula et al., 2016a).

In Africans, there are currently few studies that have evaluated the impact of genetic variation in *CES1* and *NOS3* on enalapril response or the likelihood of being diagnosed with RHTN. Given the crucial role of these enzymes in metabolism or response to enalapril and in BP regulating pathways, we hypothesize that they could be of pharmacogenomic importance in African populations, conferring susceptibility to phenotypes associated with poor drug response such as RHTN (Dudenbostel et al., 2016; El Rouby and Cooper-DeHoff, 2015). Thus, our study aimed to evaluate the role of genetic variation in *CES1* and *NOS3* in susceptibility to RHTN as well as estimate the baseline frequency of *CES1* CNV among African and Mixed Ancestry (MA) (i.e., admixed populations of Southern Africa resulting from intermarriages between African, European, San or Asian populations) populations of South Africa. Understanding the pharmacogenetic variants associated with susceptibility to RHTN has the potential to enhance treatment and control of hypertension among African populations.

## Materials and methods

### Study design and participants

This was a retrospective, matched case-control study, enrolling participants attending a tertiary-level Hypertension Clinic at Groote Schuur Hospital in Cape Town, South Africa (33.9413° S, 18.4622° E). This clinic provides specialised care for patients referred for the management of hypertension, including drug response phenotypes such as RHTN. Participants were classified as either poor responders (cases) or good responders (controls) to antihypertensive therapy, based on them having either RHTN or non-RHTN respectively, and matched according to age, sex, and ethnicity in a 1:1 ratio. RHTN was defined as (i) BP > 140/90 mmHg on 3 or more antihypertensive drugs including a diuretic in optimal doses and (ii) BP < 140/90 mmHg on 4 or more antihypertensive drugs in an adherent patient. Where non-adherence was suspected, it was ruled out by monitoring of amlodipine levels as described by with Jones and colleagues (Jones et al., 2017). Non-RHTN was defined as BP < 140/90 mmHg or BP > 140/90 mmHg on less than 3 antihypertensive drugs with no additional clinical indications of true resistant hypertension (Dudenbostel et al., 2016). BP and the number of antihypertensive drugs that the patients were taking were evaluated using information from the most recent visit at the time of recruitment. To ensure accurate BP measurements, an average of 6 BP readings were recorded. If patients had borderline BP (=140/90 ± 5 mmHg) at the most recent visit, an average of BP measurements from prior visits were taken into account, when we were classifying these patients into cases (RHTN) or controls (non-RHTN). The inclusion criteria were participants (i) with a confirmed diagnosis of primary or essential hypertension (defined as hypertension without any known underlying cause), (ii) of African heritage (defined as individuals of African or MA descent), (iii) on at least one antihypertensive drug for at least a year prior, and (iv) > 18 years at the time of recruitment. The exclusion criteria were participants (i) with a confirmed diagnosis of secondary hypertension (defined as hypertension with a known underlying cause), (ii) who were pregnant at the time of recruitment, (iii) with confirmed white coat hypertension or non-adherence, and (iv) not on treatment with any antihypertensive drugs.

### Sample size calculation

The sample size was calculated according to Naing et al. (2022), ensuring over 80% power. Briefly, the prevalence of RHTN was taken to be 12.6% as reported by Moosa et al. (2016) and an alpha level of 0.05 was used. This resulted in a required sample size of at least 163 participants with RHTN. Since this was a case-control study by design, 163 participants with non-RHTN would be required to serve as the control group. This resulted in a total sample size of at least 326 participants. However, we incorporated an allowance of at least 15% more participants in each group, to account for potential sample quality issues during genetic characterization. Therefore, our realized sample size was brought to a total of 389 participants, including 190 with RHTN (cases) and 189 with non-RHTN (controls). Although our study was sufficiently powered overall, our cohort was not homogenous as it was made up of African (N = 110) and MA (N = 279) participants. The African group in our study is made up of native Africans predominantly of Xhosa origin, while the MA group is made up of individuals resulting from intermarriages between native Africans and/or European, San or Asian populations (i.e., Cape Coloureds). Thus, to resolve the genetics of the two distinct ethnic groups, subgroup analyses according to ethnicity were performed. However, we acknowledge that the number of African participants, could have limited the power to detect statistically significant associations in this group.

### DNA sample preparation

DNA was extracted from 2 mL of venous blood provided by consenting participants using the Chemagic 360^®^ automated nucleic acid extraction system (Chemagen Technologies GMBH/Perkin Elmer Incorporated, United States) according to the manufacturer’s protocol. The extracted genomic DNA was evaluated for quality and quantity using electrophoresis on a 1% (w/v) agarose gel and spectrophotometry on a NanoDrop c1000 UV spectrophotometer (Thermofisher Scientific Corporation, Massachusetts, United States) respectively. DNA of good quality and quantity (at least 50 ng/μL) was used for genetic characterization.

### Selection of candidate pharmacogenomic markers in *CES1* and *NOS3* for genetic characterization

The selection of SNPs in *CES1* and *NOS3* was guided by querying several pharmacogenomic databases such as the Clinical Pharmacogenetics Implementation Consortium (CPIC) database, Food and Drug Administration (FDA) Table of Pharmacogenomic Biomarkers in Drug Labelling, Pharmacogenomics Knowledge Base (PharmGKB) database and surveying available literature. Variants were prioritised according to (i) annotation in any of the pharmacogenomic databases, (ii) predicted or known functional effect, (iii) minor allele frequency (MAF) > 0.05 in African populations and (iv) published evidence of potential clinical relevance. Furthermore, variants not previously reported in Africans but were reported to influence response/efficacy to enalapril or any other ACE inhibitor were also prioritised, to pronounce on their role in Africans as well. This strategy yielded the following variants for analysis: *NOS3* rs1799983G>T (priority 1), rs2070744C>T (priority 1), rs3918188C>A (priority 2)*, CES1* rs2244613G>T (priority 2) and *CES1* CNV (priority 3) (Table 1).

**TABLE 1:** Rationale for selection of potential candidate pharmacogenomic biomarkers in *CES1* and *NOS3* for analysis.

SNP/Variant	Functional effect	MAF/frequency in Africans^a^	Database annotation^b^	Rationale for inclusion	Literature evidence	Assigned priority level for this study^c^
*CES1* rs2244613G>T	Intron variant	0.09	PharmGKB (level 3)	MAF >0.05, variant may influence splicing and linked to ACE inhibitor response with strong pharmacokinetic evidence	Ikonnikova et al. (2022a), Ikonnikova et al. (2022b), Seychev et al. (2020)	1
*CES1* CNV	Gene deletion or duplication	—	—	Frequency unknown in Africans, structural variant associated with reduced transcriptional activity, not widely explored in Africans, but of potential pharmacogenomic significance	Yoshimura et al. (2008), Fukami et al. (2008)	3
*NOS3* rs3918188C>A	Intron variant	0.07	—	MAF >0.05, linked to enalapril response, not widely explored in Africans, but associated with variable levels of nitric oxide in Brazilians of African descent	Metzger et al. (2013), Oliveira-Paula et al. (2016a)	2
*NOS3* rs1799983G>T	Missense variant (Gly298Asp)	0.12	PharmGKB (level 3)	MAF >0.05, associated with reduced NOS3 availability, activity and low nitric oxide levels, linked to enalapril or ACE inhibitor response	Oliveira-Paula et al. (2016a), Oliveira-Paula et al. (2016b), Joshi et al. (2007)	1
*NOS3* rs2070744C>T	Promoter variant (regulatory effect)	0.18	PharmGKB (level 3)	MAF >0.05, regulates transcription, associated with differential expression of *NOS3,* linked to ACE inhibitor response	Oliveira-Paula et al. (2016b), Silva et al. (2013)	1

^a^ Minor allele frequency (MAF) obtained from Ensembl Genome Browser.

^b^ Databases accessed for gene or variant annotations: CPIC, FDA Table of Pharmacogenomic Biomarkers or PharmGKB.

^c^ Priority level 1: Annotation in either database + MAF >0.05 + strong functional effect (determined by type of variant and supported by functional studies) + literature evidence. Priority level 2: MAF >0.05 + functional effect + literature evidence. Priority level 3: literature evidence only.

### Genetic characterization

Genetic characterization was achieved through polymerase chain reaction restriction fragment length polymorphism (PCR-RFLP), quantitative PCR and validated through Sanger sequencing. Forward and reverse primers flanking regions containing *NOS3* rs1799983G>T, rs2070744C>T, rs3918188C>A and *CES1* rs2244613G>T SNPs were designed using the NCBI-Primer Blast tool [https://www.ncbi.nlm.nih.gov/tools/primer-blast/(last accessed on 10 February 2025)], Integrated DNA Technologies (IDT) Oligo Analyzer tool [http://www.idtdna.com/analyzer/Applications/OligoAnalyzer/ (last accessed on 10 February 2025)] and synthesized by Inqaba Biotechnical Industries (Muckleneuk, Pretoria, South Africa). Forward and reverse primer sequences, annealing temperatures and PCR product sizes are shown in Supplementary Table S1.

All PCRs were performed in a 25 µL reaction volume containing 100 ng genomic DNA; 1X Green GoTaq Flexi^®^ Reaction Buffer (Promega Corporation, Madison, WI, United States); 0.4 µM of deoxynucleotide triphosphates (dNTPs) (Promega Corporation, Madison, WI, United States); 1.5 mM magnesium chloride (Promega Corporation, Madison, WI, United States); 0.2 µM each of forward and reverse primers (Inqaba Biotechnical Industries (Pty) Ltd., South Africa), 1 U of GoTaq Flexi^®^ DNA Polymerase (Promega Corporation, Madison, WI, United States) and nuclease-free water. Amplification was done using a SimpliAmp^™^ Thermal Cycler (Applied Biosystems™, Thermofisher Scientific, Massachusetts, United States). The reaction conditions were an initial denaturation at 94°C for 3 min; followed by 35 cycles of further denaturation at 94°C for 30 s; annealing at temperatures specific for each SNP (Supplementary Table S1) for 30 s; initial extension at 72°C for 30 s; and final extension at 72°C for 10 min.

PCR products from amplification of regions flanking the *NOS3* rs1799983G>T and *CES1* rs2244613G>T SNPs, were digested using *BanI* and *AlwN1* restriction enzymes, respectively (New England BioLabs^®^, Ipswich, United Kingdom). In each 30 µL digest reaction, 10 µL of PCR product, 1X CutSmart™ Buffer (New England BioLabs^®^, Ipswich, United Kingdom), 3 U of restriction enzyme specific for each SNP, and nuclease-free water were added. Restriction enzyme digest reactions were incubated and inactivated on a SimpliAmp^™^ Thermal Cycler (Applied Biosystems™, Thermofisher Scientific, Massachusetts, United States) at temperatures and periods specific for each enzyme according to the manufacture’s protocol [https://nebcloner.neb.com/#!/redigest (last accessed on 10 February 2025)] and resolved using electrophoresis on a 3% (w/v) agarose gel. Restriction enzyme digestion yielded DNA fragments corresponding to the genotype of the samples as shown in Supplementary Table S2.

Commercially available TaqMan™ allelic discrimination and copy number variation (CNV) assays were purchased from Thermofisher Scientific Incorporation (Massachusetts, United States) for genotyping *NOS3* rs2070744C>T (C__15903863_10), rs3918188C>A (C__29193459_10) SNPs and for determination of *CES1* copy number (Hs00139541_cn). Each TaqMan allelic discrimination assay reaction was set up in a 10 µL reaction volume containing 5 µL of 2X TaqPath^™^ ProAmp^™^ Master Mix (Thermofisher Scientific Corporation, Massachusetts, United States), 0.5 µL of 20X TaqMan™ genotyping assay specific for each SNP and 45 ng of genomic DNA. Assays were carried out on a CFX96 Touch Real Time PCR Thermal Cycler (Bio-Rad Laboratories, Inc., California, United States). The reaction conditions were initial denaturation at 95°C for 5 min followed by 50 cycles of further denaturation and annealing at 95°C for 15 s and 60°C for 1 min respectively, then final elongation at 60°C for 30 s.

The TaqMan™ CNV assay targeted intron 11 of the *CES1* gene on chromosome 16q12.2 (assay reference genome location: chr.16:55,810,581 on build GRCh38). The reference gene was the gene encoding ribonuclease P located on chromosome 14q11.2 and the VIC dye labelled TaqMan™ CNV RNase P assay (catalogue number: 4,403,326) served as the reference assay (assay reference genome location: chr.14:20,343,370 on build GRCh38). Each reaction was carried out in a total reaction volume of 10 µL containing 5 ng of genomic DNA, 0.5 µL of RNase P assay, 0.5 µL of TaqMan CNV assay (Hs00139541_cn), 5 µL of 2X TaqPath^™^ ProAmp^™^ Master Mix and 3 µL of nuclease-free water. The reactions were carried out on the QuantStudio™ 7 Flex Real-Time PCR System (Applied Biosystems™, Massachusetts, United States) and the reaction conditions were initial denaturation at 95°C for 10 min followed by 40 cycles of further denaturation and annealing at 95°C for 15 s and 60°C for 1 min respectively.

Post-PCR clean-up using exonuclease I (*ExoI*) and thermosensitive alkaline phosphatase (*FastAP* ^*™*^) enzymes from Thermofisher Scientific (Massachusetts, United States) was done as per manufacturer’s protocol prior to Sanger sequencing. Sanger sequencing was done to validate genotyping. Each sequencing reaction was set up in a reaction volume of 10 μL, containing 5 µL of PCR product, 2 µL of BigDye™ Terminator v3.1 5X sequencing buffer, 2 µL of BigDye™ Terminator 3.1 Ready Reaction Mix (Thermofisher Scientific, Massachusetts, United States) and 1 µL of sequencing primer (Supplementary Table S1). Sequencing reactions were carried out on a SimpliAmp™ Thermal Cycler (Applied Biosystems™, Massachusetts, United States). The sequencing reaction cycling conditions and post-sequencing clean-up using EDTA/ethanol precipitation were done according to the manufacturer’s protocol (Thermofisher Scientific, Massachusetts, United States). Sequencing reactions were resolved using capillary electrophoresis on a SeqStudio^®^ Genetic Analyzer (Thermofisher Scientific, Massachusetts, United States) and analyzed using DNAStar-SeqMan Pro Sequence Assembly software (DNAStar^®^, Madison, WI).

### Statistical analyses and *in silico* functional annotation

Statistical analyses were performed using various packages in R statistical software (version 4.4.1, 2024–06–14, Vienna, Austria). Genotype, allele and haplotype frequencies, as well as linkage disequilibrium (LD) parameters were computed using SHEsis online software (Shen et al., 2016). Calculations of *CES1* gene copy number was done using CopyCaller software for Windows (Version 2.0, Applied Biosystems, Massachusetts, United States) which assigned a quality metric to the copy numbers generated for each sample, incorporating absolute *Z*-scores (±1) and confidence scores (0%–100%). Copy number calls with a confidence metric >50% were included in the final analysis. Categorical data were presented as N (%) [where N = the number of individuals in that category and % = the frequency of individuals within that category]. Continuous data were presented as mean ± standard deviation (SD) or median ± interquartile range (IQR) [25th – 75th percentile] depending on the distribution of the data. The Shapiro-Wilks test was used to assess any deviation from normality, and the Hardy-Weinberg Equilibrium (HWE) was determined for each SNP for African and MA population groups using the Chi-square test with one degree of freedom. Continuous data were compared between cases and controls either using the T-test or the Mann-Whitney U-test (Wilcoxon rank-sum test) for normally and non-normally distributed data, respectively. Categorical data were compared between cases and controls using Pearson’s Chi-square test or Fisher’s exact test.

The main outcome of interest was the association of selected polymorphisms with RHTN. Therefore, the additive model of inheritance was assumed to estimate associations with RHTN. To account for the effect of any confounding variables on the association of genetic factors with RHTN, non-genetic predictor variables (clinical or demographic factors) were screened as univariate and those with a P ≤ 0.2 were retained for analysis as covariates (altogether). A multivariable logistic regression model was fitted, to estimate adjusted odds ratios (aORs) and 95% confidence intervals (CIs) for the association between each genetic variable and RHTN using the generalised linear model (glm (.)) function in R studio. The model included age (in years), smoking status (yes/no), plasma aldosterone concentration (pmol/L), diabetes mellitus (yes/no), dyslipidaemia (yes/no), use of diabetic medications (yes/no), lipid-lowering therapy (yes/no), *CES1* CNV, *CES1* rs2244613G>T, *NOS3* rs1799983G>T, *NOS3* rs2070744C>T and *NOS3* rs3918188C>A. Adjusted P-values reflected these covariate adjustments, aORs described the strength of associations and 95% confidence intervals (CI) were used as precision estimates. Statistical significance was set at P < 0.05. Bonferroni correction was applied to correct for multiple comparison, although its use is contested. The Bonferroni correction was applied for the 4 SNPs with significant P < 0.0125 (0.05/4).

Finally, intronic SNPs found to be significantly associated with RHTN were further queried in HaploReg v4.2 (Broad Institute, Massachusetts, United States) developed by ENCODE (Ward and Kellis, 2012) to further assess their mechanistic role including potential regulatory function and any other predicted SNPs appearing in high LD in Africans.

## Results

### Study participant characteristics

A total of 379 participants (29% African and 71% MA populations) were enrolled, and their demographic and clinical characteristics are presented in Table 2. Among the participants, sex and age were similar between cases and controls, in the African and MA groups. Compared to the control group, patients with RHTN (or cases) were more likely to have been active or past smokers (MA group only), on more antihypertensive drugs (i.e., amlodipine, hydrochlorothiazide, atenolol, enalapril, spironolactone or losartan); had left ventricular hypertrophy (LVH) and higher aldosterone levels. Furthermore, there were more cases diagnosed with chronic kidney disease (CKD) (P < 0.001), stroke (P = 0.04) and on statin or other lipid lowering therapy (MA group only, P = 0.001) than controls (Table 2).

**TABLE 2:** Demographic and clinical parameters compared among participants with resistant hypertension (cases) and non-resistant hypertension (controls) for African and Mixed Ancestry groups.

African (N = 110)				Mixed ancestry (N = 279)		
Parameter	Cases (N = 58)	Controls (N = 52)	P Value	Cases (N = 132)	Controls (N = 137)	P Value
Age years	44.2 (38.3–50.0)	41.4 (37.3–44.7)	0.08	44.6 (38.1–58.1)	42.9 (37.3–53.9)	0.17
Female sex	30 (51.7%)	22 (42.3%)	0.35	72 (54.5%)	80 (58.4%)	0.61
Alcohol consumption	9 (15.5%)	15 (28.8%)	0.10	36 (27.2%)	32 (23.4%)	0.48
Smoking (Active/Past)	5 (8.6%)	20 (38.5%)	<0.001	58 (43.9%)	44 (32.1%)	0.04
Hypertension in 1st degree relatives	35 (60.3%)	31 (59.6%)	0.99	88 (66.7%)	90 (65.7%)	0.97
Left Ventricular Hypertrophy						
Yes	26 (44.8%)	20 (38.5%)	0.56	63 (47.7%)	32 (23.4%)	<0.001
No	32 (55.2%)	32 (61.5%)		69 (52.3%)	105 (76.6%)	
Aldosterone (pmol/L)	247 (125–420)	185 (117–367)	0.31	290.5 (181–449)	201 (129–306)	0.001
Comorbidities						
Chronic Kidney Disease (CKD)	14 (24.1%)	6 (11.5%)	0.14	22 (16.7%)	4 (2.9%)	<0.001
Diabetes Mellitus (DM)	7 (12.1%)	2 (3.8%)	0.17	26 (19.7%)	16 (11.7%)	0.07
Dyslipidaemia	7 (12.1%)	2 (3.8%)	0.17	23 (17.4%)	13 (9.5%)	0.05
Ischaemic Heart Disease (IHD)	4 (6.9%)	4 (7.7%)	0.99	13 (9.8%)	5 (3.6%)	0.05
Previous Stroke	1 (1.7%)	1 (1.9%)	0.99	12 (1.5%)	4 (2.9%)	0.04
Concomitant Drugs						
Lipid-lowering Therapy (e.g., statin)	18 (31.0%)	11 (21.2%)	0.28	57 (43.2%)	33 (24.1%)	0.001
Diabetic Treatment	4 (6.8%)	2 (3.8%)	0.68	19 (14.4%)	12 (8.8%)	0.18
Analgesics	5 (8.6%)	4 (7.7%)	0.99	7 (12.9%)	6 (4.4%)	0.78
Antihypertensives Drugs***						
Amlodipine	52 (89.7%)	34 (65.4%)		119 (90.2%)	80 (58.4%)	
Hydrochlorothiazide	43 (74.1%)	33 (63.5%)		104 (78.7%)	73 (53.3%)	
Atenolol	41 (70.7%)	7 (13.5%)		81 (61.4%)	20 (14.6%)	
Enalapril	48 (82.8%)	19 (36.5%)		101 (76.5%)	68 (49.6%)	
Spironolactone	23 (39.7%)	1 (1.9%)		29 (22.0%)	5 (3.6%)	
Losartan	6 (10.3%)	2 (3.8%)		33 (25.0%)	12 (8.8%)	

P-Value: significance level.

***the antihypertensive drugs are used in multiple different combinations, and therefore the statistics of the distribution was not considered, but only the prevalence/frequency of their use in our population.

### *CES1* copy number variation

A total of 372 participants included in the final analysis had *CES1* copy number calls with confidence scores >50%. The number of copies of the *CES1* gene ranged from 2–4 (Table 3). No complete deletion (<2) was observed in our cohort. Copy number 2 was the most frequent in both African (84%) and MA (64%) participants, with a statistically significant difference (P = 0.002) between the two ethnic groups. Copy number 3 was more frequent in MA individuals (33%) compared to Africans (15%) (P = 0.004). No significant difference was observed for copy number 4 (P = 0.34) between African and MA groups. Comparing to non-African populations, there were statistically significant differences (P < 0.05) in frequencies of copy number calls between the African group and the Russian (Ikonnikova et al., 2022b) and Chinese Han (Chen et al., 2021) populations. The copy number calls for the MA group were similar to either the Russian or Chinese Han populations with no statistically significant differences (P > 0.05) observed (Table 3).

**TABLE 3:** Frequencies of *CES1* copy number calls by ethnicity or population group.

Copy Number	This study		Non-African	
	African *N* (freq.)	Mixed ancestry *N* (freq.)	Russian *N* (freq.)^a^	Chinese Han *N* (freq.)^b^
1	—	—	—	73 (0.12)
2	92 (0.84)	169 (0.64) ^α^	194 (0.68) ^β^ ^µ^	409 (0.68) ^γ^ ^δ^
3	16 (0.15)	86 (0.33) ^ε^	77 (0.27) ^ζ^ ^θ^	132 (0.22) ^η^ ^κ^
4	1 (0.01)	8 (0.03) ^π^	14 (0.05) ^ρ^ ^ψ^	26 (0.04) ^σ^ ^ϕ^
5	—	—	—	2 (0.003)
Total	109 (1.00)	263 (1.00)	286 (1.00)	606 (1.00)

*CES1* copy number frequencies for non-African populations obtained from.

^a^ Ikonnikova et al. (2022b) and.

^b^ Chen et al. (2021).

Comparison of CES1 CNV, across populations.

1. Copy number 2: ^**α**^ African vs. Mixed Ancestry: P = 0.002; ^β^ African vs. Russian: P = 0.001; ^γ^ African vs. Chinese Han: P = 0.0006; ^µ^ Mixed Ancestry vs. Russian: P = 0.38; ^δ^ Mixed Ancestry vs. Chinese Han: P = 0.35.

2. Copy number 3: ^ε^ African vs. Mixed Ancestry: P = 0.004; ^ζ^ African vs. Russian: P = 0.01; ^η^ African vs. Chinese Han: P = 0.09; ^θ^ Mixed Ancestry vs. Russian: P = 0.14; ^κ^ Mixed Ancestry vs. Chinese Han: P = 0.09.

3. Copy number 4: ^π^ African vs. Mixed Ancestry: P = 0.34, ^ρ^ African vs. Russian: P = 0.0006; ^σ^ African vs. Chinese Han: P < 0.0001; ^ψ^ Mixed Ancestry vs. Russian: P = 0.27; ^ϕ^ Mixed Ancestry vs. Chinese Han: P = 0.38.

### No association of *CES1* copy number variation with resistant hypertension

The distribution of *CES1* copy numbers between cases and controls for the African and MA groups and associations with RHTN after adjusting for potential confounding variables (i.e., age, smoking, aldosterone levels, diabetes mellitus (DM), dyslipidaemia, diabetic treatment and lipid lowering therapy) are shown in Supplementary Table S3. There were no statistically significant differences in the distributions of each copy number between cases and controls (P > 0.05) for both African and MA groups. Similarly, no significant associations with RHTN were observed. *CES1* copy numbers were further categorized into copy number neutral (=2) and copy number gain (>2) as previously described by Chen et al. (2021). We observed no significant differences in the distribution of participants who were copy number neutral or had a copy number gain between cases or controls indicating no significant association with RHTN (Supplementary Table S3).

### Association of single nucleotide polymorphisms in *CES1* and *NOS3* with resistant hypertension

Genotype frequency distributions between cases and controls, and associations with RHTN for the *CES1* rs2244613G>T, *NOS3*rs1799983G>T, rs2070744C>T and rs3918188C>A SNPs in the African and MA groups after adjusting for potential confounding variables are shown in Table 4. *NOS3* rs3918188A/A genotype carriers were significantly more frequent among the controls compared to cases in the MA group and associated with reduced risk of RHTN [17% vs. 6% respectively (P = 0.0009; aOR: 0.13; CI: 0.04–0.41)]. No statistically significant differences in the distribution of variant alleles (Supplementary Table S4) and genotypes (Table 4) between cases and controls for *NOS3* rs1799983G>T, rs2070744C>T and *CES1* rs2244613G>T for both African and MA groups were observed.

**TABLE 4:** Comparison of genotype frequency distributions between resistant hypertension (cases) and non-resistant hypertension (controls) participants among African and Mixed Ancestry groups and associations with resistant hypertension.

SNP	Genotype	African group				Mixed ancestry group			
		Cases (N = 58)	Controls (N = 52)	aOR [95%CI]	P-value	Cases (N = 132)	Controls (N = 137)	aOR [95%CI]	P-value
*CES1* rs2244613	T/T	37 (0.64)	35 (0.67)	Ref.		64 (0.49)	76 (0.56)	Ref.	
	G/T	17 (0.29)	15 (0.29)	1.42 [0.32–6.61]	0.65	57 (0.43)	50 (0.37)	1.41 [0.71–2.83]	0.33
	G/G	4 (0.07)	2 (0.04)	3.69 [0.25–11.2]	0.37	11 (0.08)	11 (0.08)	0.87 [0.25–2.90]	0.81
*NOS3* rs1799983	G/G	49 (0.85)	48 (0.92)	Ref.		86 (0.65)	93 (0.68)	Ref.	
	G/T	8 (0.14)	4 (0.08)	2.20 [0.25–26.50]	0.49	38 (0.29)	41 (0.30)	0.61 [0.28–1.31]	0.21
	T/T	1 (0.02)	0 (0.00)	2.74 [0.10–68.72]	0.99	8 (0.06)	3 (0.02)	1.11 [0.23–6.41]	0.90
*NOS3* rs2070744	T/T	43 (0.75)	44 (0.85)	Ref.		79 (0.60)	78 (0.57)	Ref.	
	C/T	12 (0.21)	7 (0.14)	2.50 [0.45–16.41]	0.30	44 (0.34)	53 (0.39)	0.91 [0.43–1.92]	0.81
	C/C	2 (0.04)	1 (0.02)	1.99 [0.10–120.5]	0.99	8 (0.06)	6 (0.04)	1.04 [0.21–6.07]	0.96
*NOS3* rs3918188	C/C	25 (0.43)	19 (0.37)	Ref.		75 (0.57)	56 (0.41)	Ref.	
	A/C	21 (0.36)	23 (0.45)	0.56 [0.13–2.17]	0.40	49 (0.37)	58 (0.42)	0.42 [0.20–0.91]	0.03
	**A/A**	12 (0.21)	9 (0.18)	0.61 [0.11–3.06]	0.55	**8 (0.06)**	**23 (0.17)**	**0.13 [0.04–0.41]**	0.0009 *

P-Value: significance level; aOR: adjusted odds ratio [adjusted for age, smoking, aldosterone, diabetes mellitus, dyslipidaemia, diabetic treatment, lipid lowering therapy], CI: confidence interval, Ref: reference. Values highlighted in bold indicate statistically significant association with RHTN and * denotes significance after Bonferroni correction for multiple comparisons at P < 0.0125.

### No association of polymorphisms in *CES1* with resistant hypertension in individuals taking enalapril only

CES1 is directly involved in the metabolism of enalapril. Thus, to further resolve the role of *CES1* in RHTN, an additional analysis step was done for African (i.e., cases: N = 48; controls: N = 19) and MA (i.e., cases: N = 101; controls: N = 68) participants that were on enalapril for associations of *CES1* polymorphisms with RHTN. Allele and genotype frequencies are shown in Supplementary Tables S4, S5 respectively. It appears that there were no statistically significant differences in the distribution of *CES1* rs2244613G>T genotypes and *CES1* copy number calls (P > 0.05) between cases and controls on enalapril in both African and MA groups, even after adjusting for confounding variables. In addition, no significant associations with RHTN were observed (Supplementary Table S5).

### Linkage disequilibrium analyses and association of *NOS3* haplotypes/diplotypes with resistant hypertension

**FIGURE 1:**
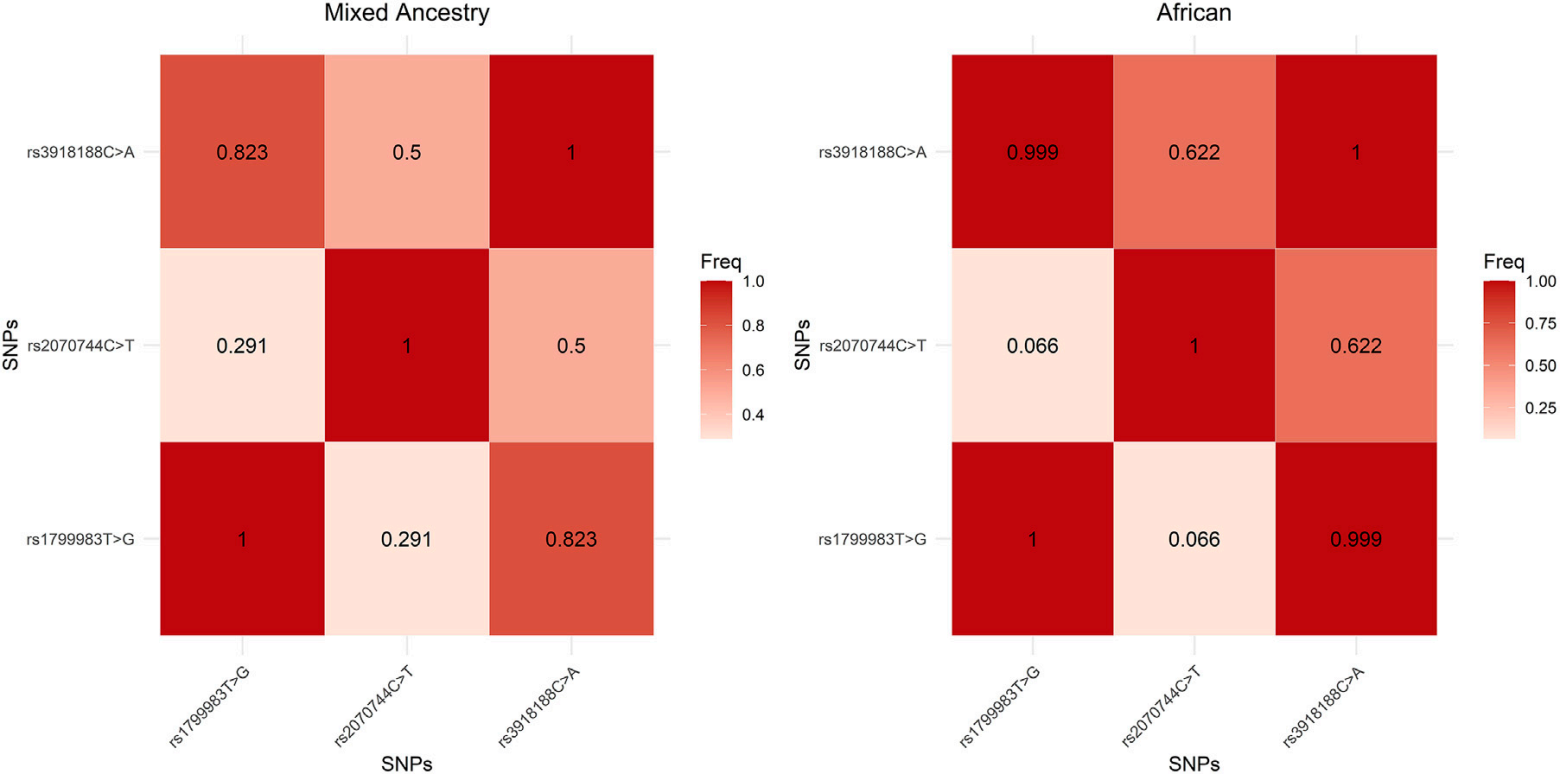
Linkage disequilibrium (LD) plots (D’) for African and Mixed Ancestry participants for NOS3 rs1799983G>T, rs2070744C>T and rs3918188C>A SNPs.

Pairwise linkage disequilibrium (LD) values for *NOS3* rs1799983G>T, rs2070744C>T and rs3918188C>A are shown in Figure 1. There is stronger allelic association between *NOS3* rs3918188C>A and rs1799983G>T (African: D’ = 0.999; MA: D’ = 0.823); and moderate allelic association between *NOS3* rs3918188C>A and rs2070744C>T (African: D’ = 0.622; MA: D’ = 0.500). The frequencies of possible haplotypes constructed from *NOS3* rs1799983G>T, rs2070744C>T and rs3918188C>A SNPs are also shown in Table 5. It seems that *NOS3* rs1799983 – rs2070744 – rs3918188 G–T–A haplotype carriers were significantly (P = 0.001) more frequent in controls (36%) than cases (24%) and were significantly associated with reduced risk of RHTN (OR: 0.54; CI: 0.37–0.78) for MA participants only. Humans are diploid and typically inherit two haplotypes-one from either parent. Therefore, to capture the full genetic variation across both copies of the *NOS3* chromosome, the frequencies of the possible haplotypes in the diploid state were determined, and associations with RHTN after adjusting for confounding variables are shown in Supplementary Table S6. It appears that participants carrying two copies of the *NOS3* rs1799983 – rs2070744 – rs3918188 G–T–A haplotype or G–T–A diplotype (G–T–A/G–T–A) were significantly (P = 0.008) more frequent in the controls (15%) than cases (6%) and associated with significantly reduced risk of RHTN in the MA group only (aOR: 0.23, CI: 0.07–0.64).

**TABLE 5:** Haplotype frequency distributions between participants with resistant hypertension (cases) and non-resistant hypertension (controls) and associations with resistant hypertension for African and Mixed Ancestry groups.

Ethnicity	Haplotype (rs1799983 - rs2070744 – rs3918188)	Haplotype frequencies				
		Combined	Cases	Controls	OR [95% CI]	P-value
African	G-C-C	21 (0.10)	13 (0.12)	8 (0.08)	1.57 [0.62–3.94]	0.34
	G-T-A	82 (0.38)	41 (0.36)	41 (0.40)	0.86 [0.50–1.50]	0.60
	G-T-C	97 (0.45)	48 (0.42)	49 (0.48)	0.80 [0.47–1.37]	0.42
	T-T-C	12 (0.06)	9 (0.08)	3 (0.03)	2.87 [0.75–10.91]	0.11
Mixed Ancestry	G-C-C	72 (0.14)	34 (0.13)	38 (0.14)	0.90 [0.55–1.49]	0.69
	**G-T-A**	**161 (0.30)**	**62 (0.24)**	**99 (0.36)**	**0.54 [0.37–0.78]**	**0.001**
	G-T-C	195 (0.37)	110 (0.42)	85 (0.31)	1.60 [1.12–2.28]	0.009
	T-C-C	46 (0.09)	24 (0.09)	22 (0.09)	1.15 [0.63–2.11]	0.64
	T-T-C	55 (0.10)	30 (0.11)	25 (0.09)	1.26 [0.72–2.22]	0.41

P-Value: significance level; OR: odds ratio, CI: confidence interval. Values highlighted in bold indicate statistically significant association with RHTN.

### In-silico functional annotation of *NOS3* rs3918188C>A

A summary of a HaploReg query for *NOS3* rs3918188C>A is presented in Supplementary Table S7. It appears that rs3918188C>A is in strong LD in Africans, with additional SNPs: rs3918181G>A, rs3918182G>A and rs3918184C>T and it coincides with chromatin features of active regulatory elements. The loci have a promoter‐like histone signature in liver (H3K4me3) and enhancer‐like marks (H3K27ac) in vascular tissue in addition to a DNAse hypersensitive site. This is consistent with regulatory potential. *NOS3* rs3918188C>A is also an expression quantitative trait locus (eQTL) for nearby genes including *KCNH2* important in BP regulation and antihypertensive drug response.

## Discussion and conclusion

We set out to investigate whether genomic variation in *CES1* and *NOS3* contributes to susceptibility to RHTN. Our findings show that *NOS3* rs3918188A allele and *NOS3* rs1799983 – rs2070744 – rs3918188 G–T–A haplotype are associated with reduced risk for RHTN in South Africans, especially among the MA ethnic group, suggesting potential implications for drug response. However, the genetic variants studied in *CES1* showed no significant association with susceptibility to RHTN.

The functional impact of the *NOS3* rs3918188C>A polymorphism remains uncertain, as it is intronic. However, there are studies, particularly in Brazilian populations of African descent (Metzger et al., 2013), linking NOS3 tagSNPs including rs3918188C>A to variable levels of circulating NO. Therefore, it is likely that rs3918188C>A potentially affects gene expression (Vaz-Drago et al., 2017), leading to changes in NO levels. In this case, increased NO levels. This is supported by HaploReg annotations (Supplementary Table S7), which place rs3918188C>A—and its African‐LD proxies (rs3918181G>A, rs3918182G>A, rs3918184C>T)—in chromatin contexts characteristic of active regulatory elements, with promoter‐associated H3K4me3 in liver and enhancer‐associated H3K27ac plus DNAse hypersensitivity in vascular tissue, suggesting allele‐dependent transcriptional modulation. *NOS3* rs3918188C>A is also an eQTL for the vascular potassium‐channel gene, *KCNH2*, and it is likely that it influences vascular excitability, providing a mechanistic basis for inter‐individual differences in baseline vascular tone. Since ACE inhibitors such as enalapril, exert part of their antihypertensive effect through NO‐mediated vasodilation, variation at this locus could also underlie the differential susceptibility to RHTN or drug response observed. Functional validation—ideally via allele‐specific reporter assays in endothelial and hepatic cells—and fine‐mapping of the LD block may be essential next steps to confirm causality.

Other SNPs, such as *NOS3* rs3918226C>T, rs743506G>A, rs1799983G>T and rs2070744C>T, have also been found to be in high LD with *NOS3* rs3918188C>A (Oliveira-Paula et al., 2016a). Our study also found *NOS3* rs1799983G>T and rs2070744C>T, to be in LD with rs3918188C>A (Figure 1), and the combination of *NOS3* rs1799983G>T - rs2070744C>T - rs3918188C>A*;* to form a G-T-A haplotype or diplotype, to be associated with reduced risk for RHTN. This shows that the reduced risk for RHTN conferred by the *NOS3* rs3918188A allele might also be as a result of allelic associations with rs1799983G>T and rs2070744C>T. The functional consequences of the *NOS3* rs1799983G>T and rs2070744C>T SNPs have been well defined in literature. For example, *NOS3* rs1799983T allele has been shown to be associated with NOS3 dysregulation and diminished NO bioavailability compared to *NOS3* rs1799983G allele (Joshi et al., 2007). *NOS3* rs2070744C allele has been shown to reduce *NOS3* promoter activity, leading to reduced NO levels compared to rs2070744T allele (Nakayama et al., 1999; Senthil et al., 2005; Nagassaki et al., 2005). Among Africans, *NOS3* rs1799983T and rs2070744C have both been shown to be associated with essential hypertension (Algerian, Moroccan and Sudanese populations, although not typical of people from Sub-Saharan Africa) (Gamil et al., 2017; Jemaa et al., 2007; Amrani et al., 2015). *NOS3* rs2070744T/T has been shown to be associated with favourable BP response to enalapril among South Africans (Swati, Ndebele, Xhosa, and Zulu ethnic groups) (Masilela et al., 2021). It appears that the *NOS3* rs1799983G and rs2070744T alleles are protective against high BP and are determinants of good BP response, respectively. Therefore, it is possible that the protective effect of the *NOS3* rs3918188A allele against RHTN observed may also be due to the additive effects of these polymorphisms.

Polymorphisms in *CES1* gene are well-documented to affect the response to ACE inhibitors. However, *CES1* rs2244613C>A and CNV did not show an association with RHTN despite evidence of associations with enalapril response in previous studies (Ikonnikova et al., 2022b). Nonetheless, we present, for the first time, the relative frequencies of *CES1* CNV in Africans. The CNV assay detected the presence of a *CES1* subtype, *CES1A2,* which has been reported to be associated with reduced transcriptional activity (Fukami et al., 2008) although findings are inconsistent (Yoshimura et al., 2008). We observed CNV patterns not previously documented in African populations, with an overall profile comparable to non-African cohorts (Table 3); the only notable difference was the absence of individuals with copy number calls of 1 and >4, which have been reported in a Chinese Han population (Chen et al., 2021). It remains possible that more diverse African groups may have a greater range of *CES1* CNV, as we only investigated South African participants of predominantly Xhosa and MA origin.

CES1 is one of the most abundant drug-metabolising enzymes in the liver (Her and Zhu, 2020; Boberg et al., 2017), whose expression levels have been reported to surpass the CYP3A4 enzyme (Boberg et al., 2017). In addition to ACE inhibitors, CES1 is involved in the metabolism of several clinically important drugs, including clopidogrel, methylphenidate, dabigatran, and simvastatin, which are frequently prescribed among Africans (WHO, 2025). CNVs at the *CES1* locus—arising mainly from non-allelic homologous recombination events between *CES1* and its nearby pseudogenes (*CES1P1* and *CES1A2*)—can alter gene dosage and potentially affect enzyme expression or activity (Rasmussen et al., 2018). The population-specific patterns observed (Table 3) likely reflect any of recombination, genetic drift, founder effects, and selection pressures from local environmental exposures (Rasmussen et al., 2018; Stage et al., 2017b). While we did not observe significant associations in this study, the pharmacogenomic relevance of *CES1* CNVs warrants further investigation. Future studies should assess allele-specific expression and protein levels by copy number, perform *in vitro* enzyme activity assays, and explore pharmacokinetic correlations to clarify the impact of *CES1* CNVs on drug response in African populations, as well as leverage other emerging functional genomics techniques (Liu et al., 2023; Zhang et al., 2024; Baysoy et al., 2024).

Our study has some limitations, the most significant being the small sample size. Although the sample size was calculated, the number of individuals belonging to the African group was relatively small in our analyses. This can be attributed to the catchment area of our recruitment site, Groote Schuur Hospital, whose surrounding areas are densely populated by individuals of MA compared to African and our study mirrors this (Statistics South Africa, 2025). Furthermore, there is evidence of differences in heath seeking behaviour among different population groups, and differences in the use of alternative complementary medicines to manage hypertension which is reportedly higher among African population groups compared to the MA (Gohar et al., 2008; Simpson, 1998). These scenarios may have limited our ability to draw definitive conclusions for the African group as access to more participants in this group was limited. Further studies with larger samples of African individuals are needed.

In conclusion, our study sheds light on the association between genetic variation and RHTN in an African population. We identified associations between *NOS3* genetic variation and reduced risk for RHTN, among the MA group. In this study, we take the opportunity to give probably the first report on *CES1* CNV distribution in an African population. Moving forward, larger prospective cohort studies involving gene expression analyses or biochemical measurements of plasma nitric oxide levels to elucidate on the functional effect of *NOS3* polymorphisms are essential, especially in African populations. In addition, non-targeted and hypothesis free approaches [i.e., genome wide association analyses (GWAS)] that would capture more variation are needed. Such efforts will not only enhance our understanding of genetic factors underlying RHTN but also pave the way for personalized treatment strategies tailored to African hypertensive patients (Katsukunya et al., 2023).

## Data Availability

The original contributions presented in the study are publicly available. This data can be found here: 10.5281/zenodo.15525258.
