# Supplementary material for: NOS3 rs3918188C>A is associated with susceptibility to resistant hypertension while CES1 genetic variation was not associated with resistant hypertension among South Africans
Source: Front Genet. 2025 Jun 4;16:1608423. doi: 10.3389/fgene.2025.1608423 (PMC12174097; doi:10.3389/fgene.2025.1608423)
Supplement: Supplementary file 1 [file Table1.docx]

**SUPPLEMENTARY TABLES**

**Table S1**: **Primer sequences, annealing conditions and PCR product sizes**

| **SNP** | **Forward Primer (5′-3′)** | **Reverse Primer (5′-3′)** | **Ta (°C)** | **PCR Product (bp)** | **Ref** |
| --- | --- | --- | --- | --- | --- |
| *CES1* rs2244613C>A | TGTCGTCTGTTCCTCCTAAG | GTTGGTTGGTCAGTTTGTTT | 58.4 | 480 | designed |
| *NOS3* rs1799983G>T | ACTTCCGAATCTGGAACAGC | ACCCAGTCAATCCCTTTGGTG | 61.1 | 529 | designed |
| *NOS3* rs2070744C>T | CCTCAGATGGCACAGAACTAC | CCTTGAGTCTGACATTAGGGTATC | 60 | 346 | designed |
| *NOS3* rs3918188C>A | TAGCCAGGAGTGAGGAAAGA | AGTCTCGCTGTGTTGTTCAG | 60 | 309 | designed |

Ta: annealing temperature, Ref: reference

**Table S2: DNA bands corresponding to genotypes after restriction enzyme digestion**

| **SNP** | **Restriction Enzyme** | **DNA band sizes corresponding to genotype** |
| --- | --- | --- |
| *NOS3* rs1799983G>T | *BanI* | G/G: 86 bp, 443 bp |
|  |  | G/T: 86 bp, 443 bp, 529 bp |
|  |  | T/T: 529 bp |
| *CES1* rs2244613C>A | *AlwN1* | G/G: 480 bp |
|  |  | G/T: 92 bp, 388 bp, 480 bp |
|  |  | T/T: 529 bp |

**Table S3: Comparison of *CES1* copy number variation between resistant hypertension (cases) and non-resistant hypertension (controls) participants among African and Mixed Ancestry groups and associations with resistant hypertension. No statistically significant associations observed.**

| **Copy**  **Number** |  | | **African group** | | | **Mixed Ancestry group** | | | | |
| --- | --- | --- | --- | --- | --- | --- | --- | --- | --- | --- |
|  | **Cases**  **(N=57)** | | **Controls**  **(N=52)** | **aOR**  **[95%CI]** | **Adjusted**  **P-Value** | **Cases**  **(N=126)** | **Controls**  **(N=137)** | **aOR**  **[95%CI]** | **Adjusted P-Value** |  |
| 2 | 49  (0.86) | | 43  (0.82) | Ref. |  | 83  (0.66) | 86  (0.63) | Ref. |  |  |
| 3 | 7  (0.12) | 9  (0.17) | | 0.99  [0.18 – 6.05] | 0.99 | 41  (0.33) | 45  (0.33) | 0.75  [0.37 – 1.48] | 0.83 |  |
| 4 | 1  (0.02) | | 0  (0.00) | 0.38  [0.02 – 9.56] | 0.56 | 2  (0.02) | 6  (0.04) | 0.31  [0.03 – 1.81] | 0.20 |  |
| Neutral  (=2) | 49  (0.86) | | 43  (0.83) | Ref. |  | 83  (0.66) | 86  (0.63) | Ref. |  |  |
| Gain  (>2) | 8  (0.14) | | 9  (0.17) | 1.27  [0.26 – 7.35] | 0.77 | 43  (0.34) | 51  (0.37) | 0.69  [0.36 – 1.33] | 0.27 |  |

P-Value: significance level; aOR: adjusted odds ratio [adjusted for age, smoking, aldosterone, diabetes mellitus, dyslipidaemia, diabetic treatment, lipid lowering therapy], CI: confidence interval, Ref: reference

|  | | **African** | | | | | **Mixed Ancestry (MA)** | | | | |
| --- | --- | --- | --- | --- | --- | --- | --- | --- | --- | --- | --- |
| **SNP** | **Allele** | **African Combined**  **(N=110)** | **Cases**  **(N=58)** | **Controls**  **(N=52)** | **P-Value** | **OR**  **[95%CI]** | **MA Combined**  **(N=269)** | **Cases**  **(N=132)** | **Controls**  **(N=137)** | **P-**  **Value** | **OR**  **[95%CI]** |
| *CES1*  rs2244613 | G | 44 (0.20) | 25 (0.22) | 19 (0.18) | 0.54 | 1.23 [0.63 – 2.39] | 151 (0.28) | 79 (0.30) | 72 (0.26) | 0.34 | 1.20 [0.82 – 1.75] |
| *NOS3*  rs1799983 | T | 14 (0.65) | 10 (0.09) | 4 (0.04) | 0.14 | 0.42 [0.13 – 1.40] | 101 (0.19) | 54 (0.21) | 47 (0.17) | 0.33 | 0.81 [0.52 – 1.24] |
| *NOS3*  rs2070744 | T | 25 (0.12) | 16 (0.14) | 9 (0.09) | 0.22 | 1.70 [0.72 – 4.05] | 125 (0.24) | 60 (0.23) | 65 (0.24) | 0.82 | 0.96 [0.64 – 1.43] |
| *NOS3*  rs3918188 | A | 86 (0.40) | 45 (0.39) | 41 (0.40) | 0.83 | 0.94 [0.55 – 1.62] | 169 (0.32) | **65 (0.25)** | **104 (0.38)** | **<0.001*** | **0.53 [0.37 – 0.77]** |
| **For only participants on enalapril, analysis for CES*1* rs2244613** | | | | | | | | | | | |
| **SNP** | **Allele** | **African Combined**  **(N=67)** | **Cases**  **(N=48)** | **Controls**  **(N=19)** | **P-Value** | **OR**  **[95%CI]** | **MA**  **Combined (N=169)** | **Cases**  **(N=101)** | **Controls**  **(N=68)** | **P-**  **Value** | **OR**  **[95%CI]** |
| *CES1*  rs2244613 | G | 28 (0.42) | 22 (0.23) | 6 (0.16) | 0.36 | 1.59 [0.59 – 4.28] | 96 (0.57) | 63 (0.31) | 33 (0.24) | 0.16 | 1.41 [0.86 – 2.31] |

**Table S4: Variant allele frequency distributions between cases and controls for African and Mixed Ancestry (MA) participants**

Values highlighted in bold indicate statistically significant association with RHTN and * denotes significance after Bonferroni correction for multiple comparisons at P < 0.0125.

**Table S5: Comparison of genotype frequency distributions between participants on enalapril with resistant hypertension (cases) and non-resistant hypertension (controls) among African and Mixed Ancestry groups.**

|  |  | **African group** | | | | **Mixed Ancestry group** | | | |
| --- | --- | --- | --- | --- | --- | --- | --- | --- | --- |
| **SNP** | **Genotype** | **Cases**  **(N=48)** | **Controls**  **(N=19)** | **aOR**  **[95%CI]** | **Adjusted P-Value** | **Cases**  **(N=101)** | **Controls**  **(N=68)** | **aOR**  **[95%CI]** | **Adjusted**  **P-Value** |
| *CES1*  rs2244613 | T/T | 30 (0.63) | 14 (0.74) | Ref. |  | 47 (0.47) | 39 (0.57) | Ref. |  |
|  | G/T | 14 (0.29) | 4 (0.21) | 1.96 [0.22 – 27.9] | 0.57 | 45 (0.45) | 25 (0.37) | 1.26 [0.53 – 2.99] | 0.60 |
|  | G/G | 4 (0.08) | 1 (0.05) | 1.16 [0.04 – 53.9] | 0.93 | 9 (0.09) | 4 (0.06) | 1.43 [0.27 – 8.44] | 0.67 |
| *CES1*  Copy number | 2 | 39 (0.81) | 17 (0.89) | Ref. |  | 66 (0.65) | 46 (0.68) | Ref. |  |
|  | 3 | 7 (0.15) | 2 (0.11) | 1.00 [0.03 – 33.3] | 0.99 | 30 (0.30) | 19 (0.28) | 0.64 [0.25 – 1.61] | 0.35 |
|  | 4 | 1 (0.02) | 0 (0.00) | 1.26 [0.05 – 32.2] | 0.98 | 2 (0.02) | 3 (0.04) | 0.70 [0.04 – 8.17] | 0.78 |
|  | Neutral  (=2) | 39  (0.81) | 17  (0.89) | Ref. |  | 66  (0.65) | 46  (0.67) | Ref. |  |
|  | Gain  (>2) | 8  (0.17) | 2  (0.11) | 1.74 [0.33 – 9.09] | 0.51 | 32  (0.32) | 22  (0.33) | 0.65 [0.26 – 1.56] | 0.33 |

P-Value: significance level; aOR: adjusted odds ratio [adjusted for age, smoking, aldosterone, diabetes mellitus, dyslipidaemia, diabetic treatment, lipid lowering therapy], CI: confidence interval, Ref: reference

| **NOS 3 Diplotype** | **Cases** | **Controls** | **P-value** | **aOR**  **[95% CI]** |
| --- | --- | --- | --- | --- |
| **African group** | | | | |
| G-T-C / G-C-A  ^α^ | 10 (0.17) | 9 (0.18) | Ref. |  |
| G-T-A / G-T-C ^β^ | 19 (0.28) | 18 (0.35) | 0.34 | 0.52 (0.13 – 2.01) |
| G-T-A / G-T-A | 29 (0.50) | 24 (0.47) | 0.18 | 0.38 (0.09 – 1.56) |
| **Mixed Ancestry group** | | | | |
| G-T-C / G-C-A ^α^ | 88 (0.67) | 79 (0.58) | Ref. |  |
| G-T-A / G-T-C ^β^ | 35 (0.28) | 38 (0.28) | 0.21 | 0.61 (0.28 – 1.31) |
| **G-T-A / G-T-A** | **8 (0.06)** | **20 (0.15)** | **0.008** | **0.23 (0.07 -0.64)** |

**Table S6: Diplotype frequency distributions between participants with resistant hypertension (cases) and non-resistant hypertension (controls) and associations with resistant hypertension for African and Mixed Ancestry groups.**

P-Value: significance level; aOR: adjusted odds ratio [adjusted for age, smoking, aldosterone, diabetes mellitus, dyslipidaemia, diabetic treatment, statin or lipid lowering therapy], CI: confidence interval, Ref: reference. Values highlighted in bold indicate statistically significant association with RHTN.

Minor diplotypes not included in Table, T-C-C/T-C-C (n=1), T-T-C/T-T-C (n=1)

^α^ Other diplotypes or haplotype combinations included : G-T-C/ (G-C-C, G-T-C), T-C-C/T-C-C and T-T-C/T-T-C.

^β^ Other diplotypes or haplotype combinations included : G-T-A/T-T-C.

**Table S7: Functional annotation of *NOS3* rs3918188C>A using HaploReg**

| **SNP of interest** | **Other SNPs in LD ^a^** | **Promoter histone marks** | **Enhancer histone marks** | **DNAse** | **Motifs changed** | **Selected eQTL hits** |
| --- | --- | --- | --- | --- | --- | --- |
| rs3918188C>A | - | LIV | VAS | IPSC, LIV | - | *ABP1, KCNH2, TMEM176A* |
|  | rs3918181G>A | - | LIV, VAS | - | GR, HP1-site factor | - |
|  | rs3918182G>A | - | LIV, VAS | - | BDP1, LUN-1 | - |
|  | rs3918184C>T | - | LIV | - | CACD_2, Klf4, Klf7, NRSF_disc4, NRSF_disc9, SP1_known4, Znf143_disc3 | - |

^a^ Variants with predicted r^2^ and D**' >** 0.80 in Africans

LIV: HepG2 Hepatocellular Carcinoma Cell Line; VAS: Umbilical Vein Endothelial Primary Cells; IPSC: iPS DF 6.9 cells
